# Decoding the Role of H19 in Cholestatic Liver Injury Using snRNA-seq, Spatial Transcriptomics, and Machine Learning-Based Disease Prediction

**DOI:** 10.21203/rs.3.rs-8339668/v1

**Published:** 2026-01-14

**Authors:** Grayson Welch Way, Xixian Jiang, Hongkun Lu, Nan Wu, Derrick Zhao, Yun-ling Tai, Sareh Bayatpour, Xuan Wang, Huiping Zhou

**Affiliations:** Virginia Commonwealth University; Virginia Commonwealth University; Virginia Commonwealth University; Virginia Commonwealth University; Virginia Commonwealth University; Virginia Commonwealth University; Virginia Commonwealth University; Virginia Commonwealth University; Virginia Commonwealth University

**Keywords:** Primary sclerosing cholangitis, Cholestasis, SPP1, Machine learning, H19, Long non-coding RNA

## Abstract

**Background:**

Primary Sclerosing Cholangitis (PSC) is a chronic obstructive biliary disease and remains a high-burden cholestatic liver disease with no approved therapies and a substantial recurrence rate following liver transplantation. The long non-coding RNA H19 (H19) has emerged as a potential driver of PSC progression, yet its cell-type-specific and spatially resolved mechanisms remain poorly defined.

**Results:**

Age- and sex-matched wild type (WT), H19 knockout (H19KO), Mdr2 knockout (Mdr2KO), and double-knockout (DKO; Mdr2KO/H19KO) mice were used. The liver tissues were analyzed using single nucleus RNA sequencing (snRNAseq) and NanoString GeoMx spatial transcriptomics to elucidate H19-dependent cellular and spatial alternations in cholestatic liver injury. Machine learning models (logistic regression, XGBoost, neural network, and random forest) were developed to generate cell-type specific disease prediction signatures and validated using the publicly available human dataset GSE243981. Both spatial transcriptomics and snRNAseq identified a disease-associated cholangiocyte subcluster that was significantly expanded in Mdr2KO mice, but markedly diminished in DKO mice, demonstrating a requirement for H19 in sustaining pathogenic cholangiocyte state. SPP1 signaling was significantly dysregulated in cholestatic liver injury and ameliorated with H19 deletion. Novel murine markers were identified, including Gm13775 (healthy hepatocytes) and Clu and Spp1 (healthy cholangiocytes), all of which were markedly downregulaed in disease. Machine learning-based, cell type-specific disease prediction models achieved AUC values > 0.87 when validated in the GSE243981 human dataset. Noteably,Spp1 expression decreased in cholangiocytes but was ectopically upregulated in hepatocytes in diseased liver, highlighting disrupted intercellular signaling network. Spatial analyses showed that *H19* deletion restored the disease-associated gene expression changes specifically within the bile duct region.

**Conclusion:**

H19 deletion mitigates cholestatic injury by suppressing pathogenic cholangiocyte states, normalizing SPP1-mediated signaling, and restoring bile-duct-localized transcriptional programs. These findings position H19 as a critical regulator of cholangiocyte-driven pathology and a potential therapeutic target in PSC.

## Introduction

Primary sclerosing cholangitis (PSC) is a chronic, progressive hepatobiliary disease characterized by biliary fibrosis, obstructive cholestasis and inflammation of the intrahepatic and extrahepatic bile ducts(1–3). The disruption of bile acid homeostasis contributes to a cascade of metabolic and immune dysregulation, as bile acids are important signaling molecules. PSC progresses along a spectrum with eventual development of end-stage liver failure(4). PSC is also associated with a markedly increased risk of malignancies, such as cholangiocarcinoma, gallbladder carcinoma, hepatocellular carcinoma, and colorectal carcinoma(5–10). In the United States, PSC is the fifth leading indication for liver transplantation(11). Furthermore, recurrence occurs in up to 30% of transplant recipients(11). Despite the severity of the disease, no proven pharmacotherapies exist, largely due to an incomplete understanding of the cellular and molecular mechanisms underlying PSC pathogenesis(11).

The long non-coding RNA H19 (H19) has emerged as an important regulator of PSC disease progression(12, 13). Previous research in our lab, and others, have shown that H19, which is normally not expressed in healthy hepatic tissues, is upregulated in PSC and exacerbates disease progression in Mdr2KO mice (the gold standard mouse model for PSC)(12, 14–19). However, the field lacks a mechanistic framework explaining how H19 reprograms specific hepatic cell populations, whether its effects are spatially restricted in the liver microenvironment, and which transcriptional networks are directly or indirectly governed by H19 in vivo. It is also unclear how diseae-assocaited cell states in mice correspond to those in human cholestatic liver disease, and whether concise molecular signatures can reliably classify cholangiocyte or hepatocyte health status across species. To address these gaps, we leveraged single-nucleus RNA sequencing (snRNA-seq) and spatial transcriptomic profiling across WT, H19KO, Mdr2KO and DKO mice. While single-cell RNA sequencing (scRNAseq) has advantages for profiling immune cells, snRNAseq offers several distinct benefits, including compatibility with frozen tissue, reduced dissociation-induced artifacts, and superior accuracy in profiling hepatocytes and cholangiocytes (20). Spatial transcriptomic technology, such as GeoMx^®^ Digital Spatial Profiler (DSP), allows transcriptomic analysis at specific regions based on histological morphology.

In our previous work, we demonstrated that genetic deletion of H19 significantly reduced liver fibrosis and slowed disease progression in Mdr2KO mice(15, 17). In this study, integration of snRNAseq and GeoMx DSP enabled us to 1) define disease-specific cholangiocyte and hepatocyte states associated with PSC, 2) map how h19 deletion reshapes these states at cellular and spatial levels, and 3) quantify the spatial restriction of H19-mediated transcriptional rescue. We further integrated publicly available human scRNA-seq and snRNA-seq datasets to evaluate cross-species conservation and built machinelearning prediction models based on minimal gene sets capable of classifying cholangiocyte and hepatocyte disease status with high accuracy.

## Materials and methods

### Animal Studies

C57BL/6J Mdr2KO mice were gifted by Dr. Daniel Goldenberg, Department of Pathology, Hadassah-Hebrew University Medical Center, Jerusalem, Israel. Maternal C57BL/6J H19^ΔExon1/+^ (H19KO) mice were generated and gifted by Dr. Karl Pfeifer at the NIH. Dr. Jian-Ying Wang at the University of Maryland (Baltimore, MD, USA) provided the H19KO mice. Mdr2KO and H19KO (DKO) mice were generated as previously described (17). The gender and age-matched littermates of WT, H19KO, Mdr2KO and DKO (female, 6-month-old) were used. Mice were housed under 12 h/12 h light and dark cycle with unrestricted access to water and standard chow *ad libitum*. All animal experiments followed institutional guidelines for ethical animal studies and were approved by VCU institutional animal care and use committee.

For GeoMx spatial transcriptomics, eight liver samples were collected from 6-month-old female mice (four Mdr2^−^/^−^ and four DKO). For single-nucleus RNA sequencing (snRNA-seq), liver tissues from 6-month-old female mice (one per genotype: WT, H19KO, Mdr2KO, and DKO) were flash-frozen in liquid nitrogen and stored at −80°C until processing.

### scRNAseq and snRNAseq data analysis

scRNAseq and snRNAseq analyses of both our dataset and the GSE243981 dataset were performed in R (version 4.3.3) utilizing the R package Seurat (version 5.1.0)(23, 24). For our mouse snRNA-seq data, nuclei with > 2.5% mitochondrial gene content were excluded. The R package SoupX (version 1.6.2) was used to remove ambient RNA contamination(21). For GSE243981, mitochondrial content filters of 5% (snRNA-seq) and 25% (scRNA-seq) were applied. Data normalization and scaling were performed using Seurat’s SCTransform function and data integration was performed utilizing Harmony, accounting for known batch variables (i.e., different experimental techniques used: scRNAseq, snRNAseq, 3’ and 5’ sequencing) following the Seurat SCTransform workflow(25). Predicted cell-cell communication were inferred using the R package CellChatv2 (version 2.1.0)(26). Pseudotime analyses for cell type progression with disease was performed using Monocle3 (version 1.4.26)(27). LASSO regression analysis was performed using the R package glmnet (version 4.1.10)(28, 29). Data visualization and statistical analyses were performed with SeuratExtend (version 1.2.5)(30). Statistical significance was determined using the MAST algorithm via Seurat’s FindMarkers function.

### Machine learning Modeling Methods

To assess the robustness and predictive accuracy of input features, four supervised machine learning models were developed and evaluated: a multilayer perceptron (MLP) neural network, an extreme gradient boosting (XGBoost) classifier, a random forest classifier, and a logistic regression model. For initial validation, models were trained and tested on mouse data using a 70/30 train–test split. For cross-species validation, each model was trained on the complete mouse dataset and subsequently tested on the entire human dataset (GSE243981). Mouse gene symbols were converted to their human orthologs using the gorth function in the gprofiler2 R package to enable translational comparison(31, 32). All models were trained to classify binary health status labels (healthy vs. diseased) and evaluated using the area under the receiver operating characteristic curve (ROC-AUC) as the primary performance metric with the R package pROC(33).

### GeoMx Data Analysis

GeoMx^®^ data analysis was performed using the StandR R package following their pipeline(34). QC was performed at both the region of interest (ROI) level and gene level to remove low-quality data. ROIs were filtered based on nuclear count, surface area, and library size using default thresholds. PCA was performed using the runPCA function from the scater package (version 1.36.0), and clustering was visualized using Uniform Manifold Approximation and Projection (UMAP)(35). Different normalization (TMM, CPM, Upper quartile) and batch correction methods (RUV4, LimmaRemoveBatch, SVA) were tested. Ultimately, TMM normalization and RUV4 batch correction were used based on PCA and cluster separation statistics. Differential gene expression (DGE) analysis was performed using the limma R package (version 3.64.3)(36). Significance was also tested and confirmed using DESeq2 (Version 1.48.1) (37).

## Results

### Identification of a disease-specific cholangiocyte cluster

Initial clustering and cell type annotation of the snRNAseq data identified 16 distinct clusters from 35,488 nuclei across all four genotypes. UMAP visualization confirmed proper integration across all genotypes and experimental techniques (Fig. 1A). Cell identity was assigned based on the expression of canonical maker genes, demonstrating high fidelity clustering (Fig. 1B). Compared to WT and H19KO, Mdr2KO and DKO mice exhibited drastic increases in cholangiocytes, fibroblasts, lymphocytes, and monocyte-derived macrophages (MdMQs) in terms of numbers and percent cell composition, accompanied by a reduction in hepatocytes and Kupffer cells, compared to their healthy counterparts (cholangiocytes, MdMQs and hepatocytes percentage of cells p < 0.05) (Fig. 1C). Subclustering of cholangiocytes identified a disease-associated cholangiocyte cluster (cluster 2) (Fig. 2A). Pseudotime trajectory analysis using monocle3 indicated that Cluster 2 corresponded to a late-stage disease state (Fig. 2B). Notably, H19 deletion in Mdr2KO mice significantly reduced both the number and percentage of cholangiocytes within this cluster (Fig. 2C). The top marker gene for cluster 2 was *Csmd1* (CUB And Sushi Multiple Domains 1), which was markedly upregulated in disease and downregulated upon H19 deletion (**Supp.** Figure 1 **and** Fig. 2D).

### Cellular pathways altered in PSC and ameliorated by H19 deletion

CellChatv2 analysis was performed to identify significant cell-cell signaling network and crosstalk interactions altered in Mdr2KO mice and to evaluate pathways modulated by H19 deletion. The total number of inferred interactions identified for WT, H19KO, Mdr2KO, and DKO were 255, 180, 573, and 438, respectively (**Supp.** Figure 2). Among the pathways examined, Spp1 (osteopontin), collagen, laminin, and FN1 (fibronectin) signaling were all significantly upregulated in Mdr2KO compared to WT and were normalized toward WT in DKO mice (p-value < 0.05) (Fig. 2A-B, **Supp.** Figures 3–5). Notably, Spp1 emerged as a major signaling pathway dysregulated in cholestatic liver injury and restored by H19 deletion. In healthy liver, *Spp1* expression is predominately observed in cholangiocytes, mediating signaling to Kupffer cells. In contrast, during disease progression, Spp1 expression shifts to mid-lobular hepatocytes, indicating altered paracrine signaling (Fig. 3C**- D, Supp.** Figure 6A-B). H19 deletion significantly reduces Spp1 expression and outgoing Spp1-mediated signaling from mid-lobular hepatocytes (p < 0.05) (Fig. 2C-D). Ligand-receptor analysis revealed that the top predicted interactions in the Spp1 pathway were Spp1-Cd44, Spp1-(Itgav + Itgb6), and Spp1-(Itga4 + Itgb1). Notably, Spp1-Cd44 signaling between cholangiocytes and Kupffer cells was present only in healthy liver and was lost in Mdr2KO mice, whereas the other Spp1 interactions were uniquely predicted in Mdr2KO mice (Fig. 4A-B).

### Diseased cholangiocyte gene prediction model and identification of novel healthy markers

Differential gene expression (DGE) analysis of subclustered cholangiocytes, combined with LASSO regression-based feature selection, identified six genes (*Clu*, *Spp1*, *Slco3a1*, *Cd44*, *Anxa3*, *Cftr*) for machine learning-based prediction modeling of cholangiocyte disease status (Fig. 4C). Among these, *Clu* and *Spp1* were strongly associated with healthy cholangiocytes, whereas *Slco3a1*, *Cd44*, *Anxa3*, *Cftr* were enriched in diseased cholangiocytes in Mdr2KO and DKO mice (adj. p < 0.001) (Fig. 4C). Three of the disease associated genes (*Slco3a1*, *Anxa3*, *Cftr*) were significantly reduced towards WT levels in DKO mice, indicating that H19 deletion ameliorates their dysregulation (Fig. 4C). A striking spatial shift was observed for *Spp1* and *Clu*, which transitioned from cholangiocyte-restricted expression in healthy liver to ectopic expression in mid-lobular hepatocytes in diseased liver, suggesting substantial rewiring of cellular identity and intercellular signaling during disease progression (**Supp.** Figure 6A–B).

### Cholangiocyte disease prediction model testing in humans

The expression patterns of six model genes (*Clu*, *Spp1*, *Slco3a1*, *Cd44*, *Anxa3*, *Cftr*) were evaluated in human cholangiocytes using the GSE243981 dataset (both scRNAseq and snRNAseq). Five of the six genes demonstrated the same significant expression trends in PSC patient cholangiocytes as observed in mice (p < 0.05), with *Cd44* as the exception when combining scRNAseq and snRNAseq datasets (Fig. 5A), but was significantly different in scRNAseq data alone (Fig. 5B). Cftr expression was significantly decreased in the combined data (sc/snRNAseq) (Fig. 5A), but did not reach significance in the scRNAseq-only analysis (Fig. 5B). These six genes were subsequently used to construct build cholangiocyte disease prediction models using four supervised machine learning algorithms: multilayer perceptron (MLP) neural network, XGBoost, random forest, and logistic regression. ROC–AUC analysis demonstrated robust performance, with AUC values > 0.92 in mouse datasets and > 0.87 in human cholangiocytes from GSE243981, supporting the translational relevance of the gene signatures and the predictive models (Fig. 5C).

### Diseased Hepatocytes gene prediction model and identification of a novel healthy marker

Hepatocytes were subclustered, zonally differentiated, and UMAPs generated to visualize proper data clustering and integration (Fig. 6A-B, **Supp.** Figure 7)(38, 39). Differential gene expression combined with LASSO regression identified six genes—*Gm13775, Spp1, C6, Cdh1, Npas2*, and *Cd74*—for construction of a hepatocyte disease prediction model using a random forest classifier, which achieved an AUC of 0.916 in mice (**Supp.** Figure 8A). Gm13775 emerged as the top-performing gene and a novel marker of healthy hepatocytes (**Supp.** Figure 8B–C). However, because no human homolog has been identified, *Gm13775* was excluded from downstream cross-species model development. A revised six-gene model (*Spp1*, *C6*, *Npas2*, *Cdh1, Cd74*, and *Hamp*) was generated for comparative analyses. In Mdr2KO hepatocytes *Spp1, C6, Npas2*, *Cdh1*, and *Cd74* were all significantly upregulated, while *Hamp* was significantly reduced relative to WT (adj. p < 0.001) (Fig. 6C). In DKO mice, *Spp1, Cdh1*, and *Cd74* were significantly decreased and *Hamp* significantly increased toward WT levels (adj. p < 0.01), indicating that *H19* deletion partially corrects hepatocyte dysregulation (Fig. 6D). CellChat v2 pathway analysis further revealed that APP signaling was significantly reduced in DKO mice (Fig. 2). Across all genotypes, the dominant ligand–receptor interaction was App–Cd74, with mid-lobular hepatocytes showing increased CD74 expression and receiving App-derived signaling in disease states (**Supp. Figure 9**).

### Hepatocyte disease prediction model testing in humans

The six genes used in the hepatocyte disease prediction model (Hamp, Spp1, C6, Cdh1, Npas2, and Cd74) demonstrated strong translational concordance in human hepatocytes from the GSE243981 dataset. All six genes showed consistent and significant expression changes in both the combined scRNAseq/snRNAseq dataset (Fig. 7A) and in the scRNAseq subset alone (Fig. 7B). When tested across four supervised machine learning algorithms, these six genes achieved AUC values > 0.869 in both mouse and human datasets, confirming their cross-species robustness and strong predictive performance (Fig. 7C).

### Deletion of H19 causes cell-type specific alterations in gene expression

Random forest modeling was performed across multiple cell types to identify gene expression patterns associated with *H19* deletion. Xist consistently emerged as the top predictive feature across all models (**Supp. Figure 10**). Although Xist/Tsix dominated feature importance, each cell type also showed distinct *H19*-dependent transcriptional signatures.

In hepatocytes, a 13 gene model (*Xist*, *Gphn*, *Fga*, *Tsix*, *Malat1*, *Ahsg*, *Apob*, *Ces1c*, *Spp1*, *Cers6*, *Cd74*, *Srsf1*, and *Hspa2*) achieved an AUC score of 0.972 for distinguishing H19 deletion from WT mice (**Supp. Figure 10**). Excluding *Xist* and *Tsix* modestly reduced performance (AUC = 0.902). In cholangiocytes, a 13 gene model (*Xist, Tsix, Csmd1, Chka, Frmd4b, Gnas, Npas2, Agmo, Ly6e, Gbp9, Sorbs3, Usp2*, and *1600012H06Rik*) achieved an AUC score of 0.934 (0.867 without *Xist* and *Tsix*). In macrophages, a 12 gene model (*Xist, Tsix, Mat1a, Gm42418, Eef1a1, Rpl13a, Gm19951, Clec2d, Wwc1, Rnf128, Ugt2b5*, and *Tnfrsf12a*) generated an AUC score of 0.861, decreasing to 0.764 without *Xist* and *Tsix.* Importantly, several genes within these models overlapped with those reversed by H19 deletion-that is, genes upregulated (or downregulated) in Mdr2KO vs. WT and reversed toward WT levels in DKO mice, indicating correction of PSC-associated dysregulation. Notable examples include *Spp1* and *Cd74* in hepatocytes, and *Frmd4b*, *Gnas*, *Chka*, *Csmd1*, and *Agmo* in cholangiocytes (**Supp. Tables. 1&2**).

### Spatial transcriptomics reveal that H19 deletion-mediated amelioration in hepatocytes is restricted to hepatocytes in close proximity to bile ducts

Spatial transcriptomics was utilized to investigate spatially defined effects of H19 deletion on hepatic gene expression. ROIs were selected to compare differences in isolated hepatocytes and hepatocytes in close proximity to bile ducts (Fig. 8A). DGE analysis of the different ROIs comparing DKO vs Mdr2KO mice yielded several common and unique significant DEGs (Fig. 8B). Common genes decreased in DKO mice vs Mdr2KO across both regions were genes associated with cellular stress responses, such as *Hspa5*, *Hspa8*, and *Hsp90aa1* (p < 0.01 across both regions), and fatty acid synthesis gene *Fasn* (p < 0.01); however, several cholesterol metabolism genes were only significantly reduced in hepatocytes neighboring bile ducts (e.g., *Apoa1*, *Apoa2*, and *Apoa5*) (Fig. 8B). Several genes related to significant disease-associated pathways (APP, Collagen, FN1, LAMININ) ameliorated by H19 deletion as well as hepatocyte-to-cholangiocyte transition marker genes (*Sox4* and *Sox9*) are only significantly reduced in regions with hepatocytes neighboring bile ducts (Fig. 8B). Four of the five hepatocyte disease-associated prediction modeling genes were only significantly ameliorated in hepatocytes neighboring cholangiocytes (*Spp1, C6, Cdh1*, and *Cd74*) (Fig. 8C), demonstrating that H19 deletion-mediated transcriptional recovery is spatially restricted to periductal hepatocytes.

## Discussion

Understanding how PSC reshapes hepatobiliary cell states at single-cell and spatial resolution has been a longstanding barrier in the field. Although H19 has been implicated in cholestatic injury, the mechanisms by which it influences specific cellular trajectories and the spatial architecture of disease have remained unresolved. (12, 14–19). In this study, supported by findings from *Andrews, et. al*, our integrative snRNA-seq, spatial transcriptomic, and machine-learning strategy addresses these gaps and provides several conceptual advances that reframe the role of H19 in PSC pathogenesis (32).

A central finding of this study is the identification of a disease-specific cholangiocyte population defined by Csmd1 and Lama4 expression that emerges during PSC progression and is substantially reduced by H19 deletion. This subpopulation aligns with a late-stage pseudotime state, establishing a mechanistic link between H19 activity and cholangiocyte trajectory convergence toward a pathogenic cell identity. The enrichment of Lama4, an extracellular matrix component with known profibrotic functions, further connects H19 biology to matrix remodeling and fibrosis.

Using multimodal approaches, we identified six genes that robustly distinguished healthy from diseased cholangiocytes across scRNA-seq and combined sc/snRNA-seq datasets. Notably, the simplest model, logistic regression, achieved strong performance, suggesting these genes define a relatively linear health vs disease axis. Several disease-specific genes (*Anxa3*, *Slco3a1*, and *Cftr*) were significantly reduced with H19 deletion (adj. p < 0.001, DKO vs Mdr2KO) (Fig. 4F). *Anxa3* encodes Annexin A3, a calcium-dependent phospholipid-binding protein elevated in liver cancers and explored as a diagnostic and therapeutic target (40, 41). *Slco3a1* (OATP3A1), a sodium-independent organic anion transporter linked to Crohn’s disease and NF-κB activation (42), also mediates bile-acid efflux and has been proposed as a protective adaptive response in cholestasis (43). Although *Slco3a1* expression was reduced in DKO vs Mdr2KO (adj. p < 0.001), it was unchanged in H19KO vs WT (p > 0.05), indicating that its down-regulation likely reflects reduced cholestasis rather than direct H19 regulation.

Two markers of healthy cholangiocytes, Clu and Spp1, showed striking disease-associated shifts: both were downregulated in cholangiocytes yet ectopically activated in hepatocytes in injured livers. Notably, reduced circulating CLU (clusterin) levels correlate with worse outcomes in biliary atresia, underscoring the clinical significance of losing these homeostatic markers (44). Despite its pathogenic role, H19 did not preserve healthy cholangiocyte identity and instead further suppressed Spp1 expression (p < 0.05). Beyond cholestatic liver disease, Spp1 signaling drives mesenchymal transition in pancreatic ductal adenocarcinoma, and its inhibition reduces tumor burden and improves survival (45). In healthy liver, cholangiocytes are the dominant source of Spp1 (46). Here, however, we find that during cholestatic injury Spp1 signaling is elevated but largely reprogrammed to originate from hepatocytes, suggesting a pathologic shift in intercellular communication. This hepatocyte-derived Spp1 may promote hepatic stellate cell (HSC) activation and myofibroblast transition, while suppression of this aberrant hepatocyte signal could limit fibrogenesis without compromising physiologic Spp1 function in cholangiocytes.

snRNA-seq analysis uncovered distinct, cell-type–specific transcriptional alterations driven by cholestatic disease and modulated by H19 deletion. Diseased hepatocytes exhibited ectopic activation of genes normally restricted to other healthy cell types, including Spp1 and Clu (cholangiocytes) and Cd74 (antigen-presenting cells). H19 deletion reversed these aberrant expression patterns: Clu and Spp1 were significantly elevated in Mdr2KO compared with WT, but markedly reduced in DKO relative to Mdr2KO (adjusted p < 0.001) (Fig. 4, Supp. Figure 6). In alignment with prior findings linking hepatic Cd74 up-regulation to Ikbkb loss, Mdr2KO hepatocytes demonstrated significantly decreased Ikbkb expression compared with WT (adjusted p < 0.001)(47). Both Cd74 induction and Ikbkb suppression were normalized in DKO hepatocytes (adjusted p < 0.001) (Supp. Table 3), indicating that H19 influences hepatocyte immune signaling pathways. Finally, we identified a previously unrecognized healthy hepatocyte marker, Gm13775, a non-coding RNA with no known homologs. Its biological role remains undefined and warrants further functional investigation.

A novel and unexpected discovery was the strong positive association between H19 and the X-chromosome inactivation regulators Xist and its antisense transcript Tsix. Xist expression was significantly reduced across all cell types in H19KO vs WT, DKO vs Mdr2KO, and combined DKO/H19KO vs Mdr2KO/WT comparisons (adjusted p < 0.001). This reduction was evident both in magnitude (log-fold change) and in the fraction of cells expressing Xist. For example, in cholangiocytes, Xist-positive cells decreased from > 70% to < 6% in H19KO vs WT and DKO/H19KO vs Mdr2KO/WT comparisons. This relationship suggests a previously unrecognized regulatory connection between H19 and the Xist–Tsix axis. Perturbation of this epigenetic network could generate pseudo–copy number–like expression shifts in females and may contribute to sex-specific transcriptional differences in cholestatic liver disease (48).Further mechanistic dissection of this interaction is clearly warranted.

We identified multiple cell type–specific differentially expressed genes influenced by H19. Machine-learning models distinguishing H19 WT from H19 knockout cells performed strongly across all major cell populations (AUC > 0.85 when including Xist), underscoring robust and highly cell type–specific transcriptional signatures. In hepatocytes, Spp1 and Cd74—two key disease-associated genes—were clearly regulated by H19, with H19 deletion restoring their expression toward WT levels. Of note, the APP–CD74 signaling axis has been implicated in promoting fibrosis in the kidney (49), raising the possibility of a conserved H19-dependent fibrogenic mechanism across organ systems. Additional hepatocyte-specific H19-responsive genes included the lipid metabolism regulators Cers6, Ces1c, and Apob, as well as Ahsg, all of which were downregulated following H19 deletion. Elevated Ahsg expression has previously been linked to tumor proliferation (50), suggesting broader relevance to liver tumorigenesis. In cholangiocytes, H19 deletion influenced several genes, including Frmd4b, Chka, Csmd1, Gnas, and Agmo. While increased Csmd1 expression has been associated with hepatocellular carcinoma (51), other studies propose that CSMD1 may function as a tumor suppressor depending on cellular and disease context (52, 53), highlighting the complexity of its role in cholangiocyte biology.

GeoMx spatial transcriptomics demonstrated that the improvement in hepatocyte disease-specific gene expression following H19 deletion was spatially restricted to hepatocytes adjacent to bile ducts. This pattern is consistent with prior reports showing that cholangiocytes are the primary source of H19 during cholestatic injury and that H19 can be transferred to neighboring cells via extracellular vesicles (14, 15, 17). Together, these findings suggest that H19-driven pathogenic signaling is mediated through local, bile duct–proximal intercellular communication, and that its removal selectively normalizes transcriptional programs in hepatocytes directly influenced by cholangiocyte-derived H19.

Taken together, these findings demonstrate that H19 deletion induces both cell-type–specific and spatially resolved transcriptomic remodeling, restoring healthy gene-expression programs across multiple hepatic lineages and attenuating PSC-related pathology in both mouse and human datasets. While these results position H19 inhibition as a compelling therapeutic strategy for cholestatic liver injury, the newly uncovered regulatory interplay between H19 and the Xist/Tsix epigenetic axis underscores the need for careful evaluation of sex-dependent and broader epigenetic consequences prior to clinical translation.

### Short comings

Although we were able to validate disease-associated differential gene-expression patterns and predictive modeling outputs using an independent human dataset, we could not directly validate the H19-deletion–specific findings due to the absence of comparable datasets. Additionally, the sequencing depth in both our mouse and publicly available human snRNA-seq datasets was insufficient to reliably detect H19, and H19 was not included in the GeoMx Whole Transcriptome Atlas (WTA) panel. These constraints limited our ability to perform direct cross-species validation of H19-dependent effects at either the single-cell or spatial resolution.

## Data Availability

All data and analysis methodology will be made available upon requests to the corresponding author. snRNAseq and spatial transcriptomic data will be uploaded to GEO.

## Abbreviations

AF: Alexa Fluor
aSMA: alpha Smooth Muscle Actin
AUC: Area Under the Curve
DEGs: Differentially Expressed Genes
DGE: Differential Gene expression
DKO: Mdr2KOH19−^/^−Double Knockout
DSP: Digital Spatial Profiler
HCC: Hepatocellular Carcinoma
H19: long non–coding RNA H19 H19KO H19 Knockout
LASSO: Least Absolute Shrinkage and Selection Operator
Mdr2: Multidrug resistance 2
PBC: Primary Biliary Cholangitis
PSC: Primary Sclerosing Cholangitis
RNA: Ribonucleic Acid
ROC: Receiver Operating Characteristic
scRNAseq: Single Cell RNA Sequencing
snRNAseq: Single Nuclear RNA sequencing
WT: Wild Type
WTA: Whole Transcriptome Atlas
